# How anxious is too anxious? State and trait physiological arousal predict anxious youth’s treatment response to brief cognitive behavioral therapy

**DOI:** 10.1186/s40359-020-00415-3

**Published:** 2020-05-12

**Authors:** Caitlyn C. McCormack, Rebekah J. Mennies, Jennifer S. Silk, Lindsey B. Stone

**Affiliations:** 1 Department of Psychology, Christopher Newport University, 1 Avenue of the Arts, Newport News, VA 23606 USA; 2 Department of Psychology, Temple University, Philadelphia, PA USA; 3 Department of Psychology, University of Pittsburgh, Pittsburgh, PA USA

**Keywords:** Pediatric anxiety, Brief CBT, Electrodermal activity, Physiological assessment, Treatment response, Sympathetic nervous system

## Abstract

**Background:**

Exposure therapy is the gold standard for treating childhood anxiety, yet not all youth improve. Children do not always have insight on their distress, which can limit the utility of self-reported units of distress (SUDS) during exposures. Physiological assessment provides an objective means of monitoring emotional arousal. Electrodermal activity (EDA) in particular indexes sympathetic nervous system arousal which is heavily linked to anxiety. The aim of the current study was to examine the feasibility and utility of incorporating EDA assessment in an in-session exposure. We examined concordance between EDA and SUDS, and whether either predicted treatment response.

**Methods:**

Thirty-four youth who met DSM-5 criteria for generalized, separation, and/or social anxiety disorder completed brief CBT (8 sessions) and completed a survey on trait physiological arousal. EDA and SUDS were collected from 18 youth (9 female, ages 9–14) during a mid-treatment exposure. Changes in anxiety severity were examined post-treatment.

**Results:**

SUDS were not correlated with trait or state physiological arousal. There was a large association between heightened sympathetic arousal and poorer post-treatment response. Similarly, SUDS indices of greater fear activation and habituation were associated with poorer post-treatment response with a small to moderate effect size. Supplemental analyses among the full sample aligned: trait physiological arousal predicted poorer treatment response.

**Conclusions:**

The lack of concordance between sympathetic arousal and SUDS indices highlights the limitations of relying solely on SUDS with pediatric populations. EDA provided unique data on youth’s distress during exposures. Thus, results indicate that physiological assessment may exhibit clinical utility for aiding clinicians in monitoring youth’s progress in exposure therapy.

**Trial Registration:**

ClinicalTrials.gov Identifier: NCT02259036.

## Background

Cognitive behavioral therapy (CBT) is recognized as a highly efficacious treatment for pediatric anxiety [23]. However, a substantial portion of youth (40–44%) do not improve in response to CBT [24, 44]. Exposure is the core component of CBT and predicts youth’s likelihood of treatment success [32, 43]. Helping clinicians maximize exposure effectiveness is a promising means of increasing treatment response.

Clinicians rely on clients’ subjective report of distress (SUDS) during exposures to gauge whether fear activation and/or within-session habituation occur. Yet, children can be unreliable reporters of their distress [38]. Children do not always understand SUDS as they frequently struggle with labeling and effectively communicating the intensity of their distress, which can lead to miscommunications during therapy [28]. Additionally, in many cases children have not yet learned to discriminate emotions and identify their anxiety based on physical cues such as racing heart or sweaty palms [27]. Incongruent ratings between children’s SUDS and behavioral and physiological indices of emotional arousal [5] highlight the limitations of relying solely on children’s SUDS and point to the need to incorporate objective assessments of emotional arousal during exposure therapy.

Multimodal assessments with subjective *and* physiological data, as an objective assessment, may provide more comprehensive evaluations of children’s emotional arousal during exposures [1]. Assessments of the autonomic nervous system have clarified alterations in pediatric anxiety disorders [41] and thus may also inform children’s treatment progress or response. However, to date most physiological assessments of emotional arousal during exposure therapy have relied on heart rate. This comes with its own limitations, however, as heart rate is a general measure of autonomic arousal impacted by both sympathetic and parasympathetic systems. Fear has long been theorized to reflect strong sympathetic activation [3], which corresponds with decreases in parasympathetic activity [31]. These inverse changes limit the interpretation of general autonomic arousal during exposures. Thus, it is not surprising that studies relying on heart rate have yielded inconsistent results. Among adults, higher heart rate during exposure has been linked with higher likelihood of treatment response [2, 8, 29, 34, 42], while other studies have failed to find an association [22, 37, 40]. Given the mixed results of heart rate measures, a more specific physiological measure of emotional arousal might provide clarity and prove to be more useful of an objective measure.

Electrodermal activity (EDA) indexes sympathetic arousal specifically, which is heavily implicated in anxiety [12], making EDA an ideal index for assessing youth’s emotional arousal (for review see [7, 11]). EDA measures activity of the eccrine sweat glands [18], thus higher EDA (or more intense sweating) indicates greater emotional arousal versus lower EDA suggests lower emotional arousal. Additionally, there are several potential benefits of incorporating EDA into exposure therapy. EDA assessments are (a) inexpensive, (b) non-invasive, (c) quick and easy to conduct, and critically, (d) data is immediately available and straightforward for clinicians to interpret in vivo. Although a couple studies have incorporated EDA assessments into exposure therapy among clinically anxious adults [34, 37], to our knowledge, the utility of EDA assessments for conducting exposures with clinically anxious youth has not been explored.

The current study provides an ecological assessment of pediatric exposure therapy that includes both subjective and objective assessments of emotional arousal. Clinically anxious youth completing brief CBT agreed to the collection of EDA data (in addition to ongoing SUDS ratings) during a mid-treatment exposure. We examined concordance between SUDS and EDA and tested whether either predicted decreases in youth’s anxiety severity post-treatment and at a two-month follow-up. Dominant theories of exposure therapy highlight the necessity of fear activation for allowing a new learning experience to occur [16, 20]. Therefore, we hypothesized that higher EDA, as the objective index of emotional arousal, would be the strongest predictor of treatment response.

## Methods

### Participants

**Fig. 1:**
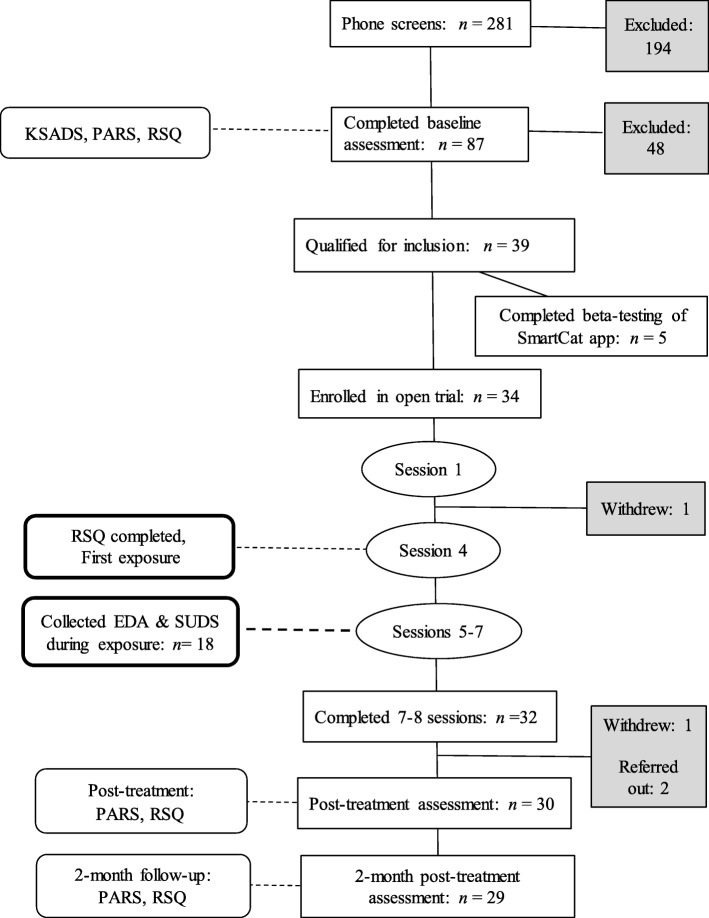
Flowchart of enrollment and data collection

Clinically anxious youth were recruited from a larger study (*n* = 34) assessing the utility of including a smartphone app in brief CBT, which consisted of 8 sessions [39]. Primary analyses focused on 18 participants who completed a mid-treatment exposure assessment. These 18 anxious youth were ages 9 to 14 (*M* = 11.38, *SD* = 1.66) who met DSM-5 [4] criteria for current Generalized Anxiety Disorder (*n* = 10), Separation Anxiety Disorder (*n* = 5), and/or Social Anxiety Disorder (*n* = 3). Of the 18 participants (50% female), 14 (77%) were European American, 3 (17%) were Biracial, and 1 (6%) was African American. Demographics are displayed in Table 1. Participants were recruited from a metropolitan city in the United States through (1) referrals from local pediatricians; (2) letters sent through a University-sponsored research registry to families interested in participating in behavioral health research studies; and (3) community advertising via flyers, internet, and print publications. Exclusion criteria included (1) neuromuscular or neurological disorder, (2) current comorbid psychiatric diagnosis that would require alternative treatment or interfere with treatment [i.e. major depressive disorder, obsessive-compulsive disorder, post-traumatic stress disorder, conduct disorder, substance abuse or dependence, or ADHD combined type or predominantly hyperactive-impulsive type], (2) a lifetime diagnosis of autism spectrum disorder, bipolar disorder, or psychotic disorder, (3) a prior trial of ≥6 sessions of CBT, (4) IQ below 70 as assessed by the Wechsler Abbreviated Scale of Intelligence (WASI: [45]) or reading level below 80 on the Wide Range Achievement Test-4 (WRAT-4: [46], 5) concurrent psychological therapy or treatment with anxiolytic or antidepressant medication (though could be on medication for ADHD if dose had been stable for at least 4 weeks), and (6) acute suicidality or risk for harm to self or others. A total of 34 youth met study criteria and completed brief CBT. The current study primarily focuses on the 18 youth who completed an additional assessment during a mid-treatment exposure (see Fig. 1). For further detail on eligibility requirements please [39].

**Table 1:** Demographics

	Exposure Assessment (*n* = 18)
Age (years) -- Mean (*SD*)	11.38 (1.66)
Female	9 (50%)
*Race or ethnic group*	
Caucasian	14 (77.8%)
African-American	1 (5.5%)
Biracial	3 (16.7%)
*Family socioeconomic status – Median*	
Family Income	100,000
*Primary anxiety disorder diagnosis*	
Separation anxiety disorder	5
Social anxiety disorder	3
Generalized anxiety disorder	10
*Anxiety Severity -- Mean (SD)*	
PARS Pre-Treatment	12.28 (3.10)
PARS Post-Treatment	7.44 (3.62)
PARS 2-month Follow-up	5.53 (3.56)

### Measures

#### Structured interviews for diagnosis and anxiety severity

Interviews were administered by trained post-doctoral fellows and clinical psychology doctoral students. Participants and their primary caregiver were individually administered the Kiddie – Schedule for Affective Disorders and Schizophrenia – Present and Lifetime Version (K-SADS-PL [26];) to determine past and current DSM-IV diagnostic status. Inter-rater reliability of anxiety diagnoses was satisfactory, κ = .77 (based on 15% of all assessments). The Pediatric Anxiety Rating Scale (PARS) was completed with the parent and child together to determine youth anxiety severity. The PARS is a clinician-rated interview that was developed to assess changes in pediatric anxiety in treatment studies, and its assessment of symptoms and severity accounts for the high co-morbidity of anxiety disorders in childhood [21]. The PARS exhibits excellent psychometric properties [36]. Anxiety severity was calculated by summing the six items assessing anxiety severity, frequency, distress, avoidance, and interference during the prior week (ICC = .91). We examined youth’s response to CBT in the current study by testing reduction in anxiety severity from pre-treatment to post-treatment and from pre-treatment to 2-month follow-up, respectively.

#### Psychophysiological assessment of emotional arousal

EDA data were collected using MindWare BioNex 3.13. Two disposable Ag/AgCl electrodes were placed on the palmar surface of the non-dominant hand. Data were sampled at 500 Hz and processed with the rolling filter. Tonic skin conductance level (SCL), which captures general changes in sympathetic arousal over time, was quantified by calculating the amount of microsiemens (or μS) occurring in 10 s segments. Data were visually inspected to ensure phasic components were detected. Because tonic-SCL did not tend to decrease or increase over time, average tonic-SCL across the exposure was computed. A log transformation was applied to normalize the data [12]. Twenty youth completed the EDA assessment. Two participants’ data were dropped due to poor data quality.

#### Subjective assessments of emotional arousal

Participants were instructed by their therapist to indicate their SUDS rating on a scale from 0 (minimum anxiety) to 8 (maximum anxiety). SUDS ratings were collected at approximately one-minute intervals starting with a baseline rating immediately prior to the start of the exposure and ending with the exposure completion. Fear activation was computed as the peak SUDS rating during the exposure (Peak-SUDS). Within-session habituation was calculated by subtracting final SUDS from Peak-SUDS, with higher scores indicating greater habituation (Habituation-SUDS).

The Responses to Stress Questionnaire (RSQ), measures voluntary and involuntary responses to stress in daily life (B. E [13].). We focus on the RSQ administered at session 4 as an index of trait physiological stress response closest in time to the exposure assessment (occurring sessions 5–7). The subscale assesses youth perception of bodily arousal during stressful events. Items from the subscale included “When I had problems, I felt it in my body (check all that apply): my heart raced, I felt hot or sweaty, my breathing sped up, my muscles got tight, none of these.” Internal consistency of this subscale in the present study was poor (α = 0.52 in the overall sample of 34 youth). Low consistency in this measure indicates youth varied in their report of perceiving different physical sensations when distressed. Although not ideal, lower internal consistency aligns with prior research supporting inconsistency in youth self-report, thus the RSQ was retained in the current study for supplemental analyses.

### Procedure

All laboratory visits (including therapy and assessments) took place in rooms designed for conducting therapy (comfortable furniture and lighting). At the outset of the baseline assessment, parental consent and child assent were first obtained. During the baseline, post-treatment, and two-month follow-up assessments, participants were administered semi-structured interviews (K-SADS, PARS, RSQ) and self-report measures. After the baseline assessment, participants completed eight (weekly) sessions of Brief Coping Cat [17]. The first three sessions consisted of psychoeducation and cognitive restructuring. The final five sessions (4–8) consisted of progressive exposures [9]. At the start of the fourth session, youth completed surveys (including RSQ) to assess anxiety prior to starting exposures in therapy.

Prior to the fifth session, participants were informed about an optional study component in which EDA would be acquired during an exposure. The collection of EDA data during mid-treatment was intentional, as initial exposures are not ideal for multiple reasons. The clinician may under-estimate the client’s ability (i.e., make the exposure too easy). Conversely, an initial exposure may be more challenging than envisioned, and simultaneously introducing additional stimuli and new people (i.e., EDA equipment and research assistant collecting data) may distract the client or amplify anxiety unnecessarily. Either scenario could limit the validity of data collected or interfere with exposure effectiveness. Participants who consented had EDA data acquired for one exposure during session 5 (*n* = 7), 6 (*n* = 10), or 7 (*n* = 1) based on laboratory availability. Exposure length varied (*M =* 6.26 min, *SD* = 2.52). Clinicians encouraged youth to engage with the feared stimulus until SUDS were equal to or lower than baseline. Youth voluntarily completed the exposure assessment without compensation. The post-treatment assessment was conducted approximately two weeks after youth completed their final therapy session. The follow-up assessment was conducted approximately two months later.

Families were compensated for their participation in baseline and post-treatment assessments (up to $360 total). Therapy was provided free of charge. Youth voluntarily completed the exposure assessment without compensation. All study aspects were approved by the Institution Review Board of the University of Pittsburgh, and the current study adheres to CONSORT guidelines.

### Data analytic plan

Multiple linear regressions tested whether Tonic-SCL, Peak-SUDS, and Habituation-SUDS predicted reductions in youth’s anxiety severity at post-treatment and at the 2-month follow-up, covarying for anxiety severity at the baseline assessment. The small sample size was underpowered to capture small and moderate effects. Therefore, in addition to reporting statistical significance we also we interpret effect sizes based on Cohen’s recommendations (*r >* .10, .30, .50 = small, moderate, and large respectively).

## Results

Bivariate correlations between SUDS and Tonic-SCL are displayed in Table 2. Self-reported indices of emotional arousal were significantly correlated: higher Peak-SUDS was associated with greater Habituation-SUDS, *r* = .50, *p* = .033. This indicates that youth who reported greater peak emotional arousal tended to report a larger decrease in distress during the exposure. In contrast, the correlations between Tonic-SCL levels and SUDS indices were quite small, indicating a lack of concordance between self-report and physiological arousal. Descriptive data on the physiological and subjective measures of emotional arousal are presented in Table 3.

**Table 2:** Bivariate associations between indices of emotional arousal and covariates during a mid-treatment exposure

	1	2	3	4	5	6	Mean	SD
1. Tonic SCL	–						9.94	4.87
2. RSQ at Session 4	0.31	–					5.64	2.06
3. Peak-SUDS	0.14	0.12	–				5.14	1.86
4. Habituation-SUDS	−0.07	−0.30	0.50^*^	–			2.55	2.06
5. Exposure length	−0.19	−0.05	0.00	−0.02	–		6.26	2.52
6. Session number	0.07	0.37	0.17	−0.20	−0.15	–	–	–
7. Age	−0.11	0.04	−0.24	−0.27	0.25	−0.20	11.38	1.66

*Note*: Tonic SCL: Mean and SD are given for non-transformed data to ease interpretation with extent literature. *RSQ* response to stress survey, physiological arousal subscale

*p < .05

**Table 3:** Qualitative analysis of data collected during a mid-treatment exposure

Age	Tonic-SCL	Peak-SUDS	Habituation- SUDS	Length	Session #
9.12	20.16	7	0	350	6
9.32	20.20	3	2	140	6
9.49	8.05	5	3	330	6
9.65	5.79	8	7	500	5
10.00	15.07	7	4	240	7
10.51	3.39	3	1	450	6
10.70	13.01	8	7	330	5
10.85	8.57	3	1	440	5
11.04	7.82	5	4	400	5
11.19	4.81	7	3	530	6
11.31	7.82	6	1	360	6
11.36	5.78	6	2	320	5
12.01	7.61	3	1	150	5
13.13	10.65	2	0	720	6
13.17	9.60	6	0	350	6
13.79	11.07	7	1	340	6
13.92	5.80	3	3	200	6
14.32	13.79	5	3	610	5

*Note*: *Tonic-SCL* tonic-skin conductance level data presented non-transformed for ease of interpretation; Habituation-SUDS reflect change in SUDS across exposure (Peak-SUDS minus final SUDS rating)

Peak-SUDS did not predict anxiety severity at post treatment, *β* = 0.08, *t*(15) = 0.31, *p* = .763, 95% confidence interval (CI): − 0.88—1.18, although there was a moderate association at the follow-up, *β* = 0.27, *t*(12) = 0.93, *p* = .373, CI: − 0.65—1.62. Although findings did not reach conventional levels of statistical significance, habituation-SUDS exhibited small to moderate associations with anxiety severity at post treatment, *β* = 0.15, *t*(15) = 0.53, *p* = .603, CI: − 0.76—1.27, and at follow-up, *β* = 0.30, *t*(12) = 0.78, *p* = .451, CI: − 0.98—2.07. Higher tonic SCL exhibited a large association with a *smaller* reduction in anxiety severity at post treatment that trended towards statistical significance, *β* = 0.46, *t*(15) = 1.91, *p =* .075, CI: − 0.91—16.65, and a moderate association at follow-up, *β* = 0.33, *t*(12) = 1.13, *p* = .282, CI: − 9.54—11.58.

Finding that *lower* sympathetic arousal was beneficial during exposure was unexpected and contradicts primary theories of exposure therapy. To address the possibility of a Type I error in this small sample, we tested another measure of physiological arousal among the full sample. We tested RSQ at session 4 as our closest proximal index of trait physiological arousal. Among the full sample, higher RSQ was moderately associated with a *smaller* reduction in anxiety severity at post treatment, *β* = 0.32, *t*(32) = 2.25, *p =* .032, CI: 0.09—1.73, and at follow-up, *β* = 0.30, *t*(25) = 1.89, *p =* .070, CI: − 0.08—1.99.

Finally, we also conducted sensitivity analyses to bolster confidence in the pattern of associations between higher physiological arousal (tonic-SCL and RSQ) and poorer treatment. First, we reran the Tonic-SCL models covarying for potential sources of variance: exposure length and child’s age. The large association between higher sympathetic arousal and poorer post-treatment response was maintained, *β* = 0.55, *t*(13) = 2.21, *p =* .045. Secondly, we considered whether results may be driven by anxiety severity pre-treatment, such that youth with more severe anxiety (higher PARS baseline) may experience higher trait physiological stress response (RSQ) and exhibit stronger state physiological arousal during exposures (heightened tonic-SCL). Pre-treatment anxiety severity was not associated with self-report of physiological arousal mid-treatment, RSQ (*n =* 34), *r* = 0.07, *p* = .672; there was a moderate association between higher pre-treatment anxiety and lower state physiological arousal: tonic-SCL (*n* = 18), *r* = − 0.38, *p* = .166.

## Discussion

We examined the utility of incorporating EDA assessment into treatment of pediatric anxiety, given the limitations of children’s subjective report. Physiological arousal was the strongest predictor of treatment response but the pattern of results were unexpected. There was a marginally significant effect whereby higher sympathetic arousal was strongly associated with *poorer* post-treatment response. Although also not statistically significant, SUDS indices yielded a consistent pattern, with modest associations between higher fear activation/ greater habituation associated with higher anxiety severity post-treatment and at follow-up. Supplemental analyses aligned: higher trait physiological arousal was a statistically significant predictor of poorer post-treatment response among the full sample.

We are hesitant to interpret the current findings as this pattern contrasts theories of exposure mechanisms [19], and evidence that higher emotional arousal is beneficial for treatment of pediatric anxiety [8, 25, 29, 33]. However, one interpretation is that physiological arousal may have particularly salient effects among youth which could mean children with high physiological arousal might not benefit from traditional exposure techniques. If the current results replicate in larger anxious youth samples, one potential clinical implication is that youth who exhibit high physiological arousal during exposures may benefit from a hierarchical approach that does not emphasize high fear activation. The biggest contribution is that results indicate that state and trait physiological arousal provide unique data on children’s emotional arousal during in-session exposure. Incorporating between-session habituation and assessments across exposures in-session may clarify how physiological arousal aids clinicians in determining youth’s progress in therapy. For example, one possibility is that youth with heightened physiological arousal may not yet have experienced between session habituation or engaging in between session exposures.

The finding that SUDS were not concordant with sympathetic arousal aligns with prior research among older adolescents and adults [5, 6, 8, 15, 30]. The overall pattern aligns with research supporting the limitations of youths’ subject report of anxiety [38]. Additionally, lack of concordance may indicate that SUDS and physiological arousal capture distinct aspects of anxiety among youth [5, 15]. Results support the utility of multimodal assessments during exposures with youth [1] and indicate that the physiological and cognitive aspects of anxiety may have different functions in anxiety maintenance and treatment among youth.

Several limitations should be noted. The small sample limited power for statistical significance, necessitating focus on effect sizes. Absence of a physiological baseline or recovery period precluded an objective index of within-session habituation, as well as our ability to discern whether EDA reflected an *increase* in state sympathetic arousal during exposure versus trait sympathetic arousal. Because the primary goal of the study was to examine the utility of incorporating a smart-phone app [39], we only examined the feasibility of collecting physiological data by during one in-session exposure. Assessments of between-session habituation, and across within-session habituation are needed since emotional arousal changes throughout treatment [10], and youth’s emotional arousal may impact their willingness to complete homework between sessions. Exposures were tailored to youth’s specific fears, not standardized, which increased ecologically validity, but this approach opens the possibility that variance across exposure types and intensity influenced results. The physiological arousal subscale of the RSQ had poor internal reliability, which threatens construct validity, but is consistent with limitations of youth’s subjective report [5, 28, 35, 38]. Given the RSQ’s subscales are supported by confirmatory factor analysis and hold across youth age and sex, the RSQ may exhibit utility for multi-dimensional analysis of youth’s response to stressors in larger samples (B. E [14];). Finally, another potentially important methodological difference is that brief-CBT is limited to 8 sessions. Full CBT protocols with youth typically include 16 sessions (e.g., [33]). Results may have differed with the full CBT protocol. Future research with larger sample sizes and standardized exposure assessments is needed to assess the utility of state and trait measures of physiological anzity in the treatment of anxious youth.

## Conclusions

In summary, physiological arousal was the strongest predictor of treatment response among our sample. Additionally, although unexpected, both state and trait high physiological arousal predicted poorer treatment response to brief CBT among the full sample. Taken together, these findings indicate that state and trait physiological arousal may impact youth’s response to brief CBT. The lack of concordancce between SUDS and indices of physiological arousal point to the benefits of incoporating objective physiological measures in exposure therapy so that clinicians have a more comprehensive assessment of youths’ anxiety. Replication among large clinical pediatric populations is needed to clarify the utility of multi-method assessment in exposure therapy for pediatric anxiety.

## Data Availability

The data that support the findings of this study are not publicly available due to ongoing analyses but are available from the corresponding author, L. Stone, upon reasonable request.

## Abbreviations

CBT: Cognitive behavior therapy
SUDS: Subjective units of distress scale/ subjective report of distress
EDA: Electrodermal activity
